# Supplementary material for: Sensory ataxia and cardiac hypertrophy caused by neurovascular oxidative stress in chemogenetic transgenic mouse lines
Source: Nat Commun. 2023 May 29;14:3094. doi: 10.1038/s41467-023-38961-0 (PMC10227029; doi:10.1038/s41467-023-38961-0)
Supplement: Supplementary file 1 — Supplementary information [file 41467_2023_38961_MOESM1_ESM.pdf]

Sensory ataxia and cardiac hypertrophy caused by neurovascular oxidative stress in chemogenetic transgenic mouse lines

Shambhu Yadav[1], Markus Waldeck-Weiermair[1], Fotios Spyropoulos[1, 2], Roderick Bronson[3], Arvind K. Pandey[1], Apabrita Ayan Das[1], Alexander C. Sisti[1], Taylor A Covington[1], Venkata Thulabandu[1], Shari Caplan[4], William Chutkow[4], Benjamin Steinhorn[1], and Thomas Michel[1,*]

[1] Division of Cardiovascular Medicine, Department of Medicine, Brigham and Women's Hospital, Harvard Medical School, 75 Francis Street, Boston, MA, 02115, USA

[2] Department of Pediatric Newborn Medicine, Brigham and Women's Hospital, 75 Harvard Medical School, Francis Street, Boston, MA, USA

[3] Department of Immunology, Harvard Medical School, Boston, MA 02115, USA

[4] Novartis Institutes for Biomedical Research, Cambridge, MA 02139

*Corresponding author: Thomas Michel, Division of Cardiovascular Medicine, Department of Medicine, Brigham and Women's Hospital, Harvard Medical School, 75 Francis Street, Boston, MA, 02115, USA. E-mail address: thomas_michel@hms.harvard.edu

Supplementary Figure 1

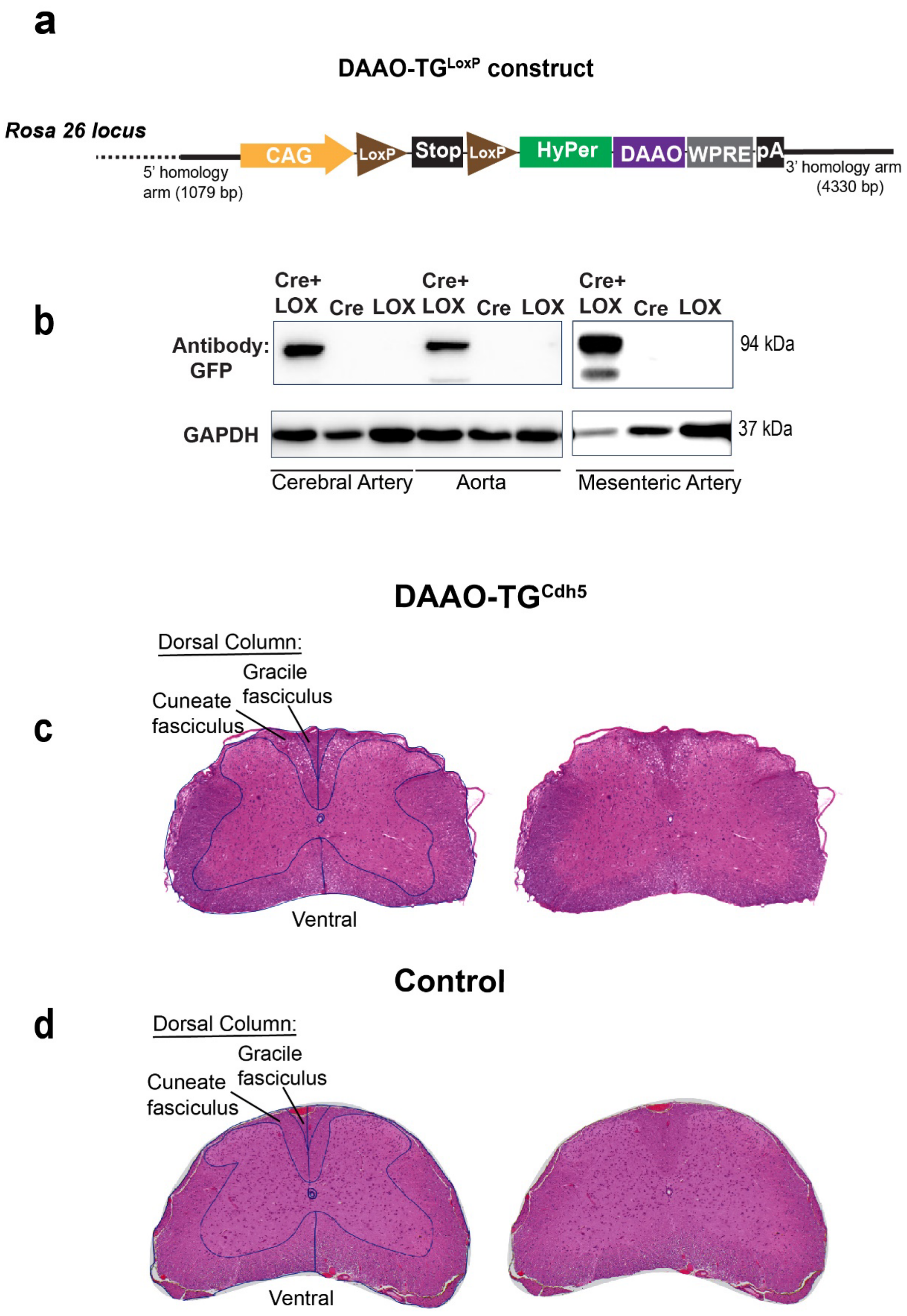

Supplementary Figure 1:

a shows a schematic representation of the construct used for transgenesis. The transgenic construct consists of the CAG promoter, followed by LoxP sites surrounding a stop codon, and then a sequence encoding a fusion protein between the HyPer (H_2O_2 ratiometric biosensor) and DAAO (yeast D-amino acid oxidase), followed by a poly(A) signal and a WPRE (Woodchuck posttranscriptional regulatory element), the entire construct flanked by homology arms (5' homology, 1079 bp and 3' homology, 4330 bp) for CRISPR/Cas9-mediated insertion into the Rosa26 locus[1]. The sequences of PCR primers used for genotyping are presented in the Methods section.

b shows an immunoblot probed for transgene expression in lysates prepared from blood vessels harvested from DAAO-TG^{Cdh5} transgenic mice (noted as Cre+Lox); from Cre-positive/transgene negative control littermates (noted as Cre); or from the parental DAAO-TG^{LoxP} mice (LOX). Lysates were probed with an antibody against GFP, which recognizes the HyPer component of the HyPer-DAAO transgenic fusion protein. n = 3 mice for each group.

c and **d** show representative haematoxylin/eosin staining of a transverse section of the lumbar spinal cord isolated from D-alanine-fed (0.75 M for 6 days) DAAO-TG^{Cdh5} (**c**) and control (**d**) mice. The left images show labels annotating the principal anatomic regions of the lumbar spinal cord, and the right images are un-annotated. In the DAAO-TG^{Cdh5} transgenic mouse, vacuoles can be seen principally in the dorsal column consistent with degeneration of the tracts in this region. These images show the full transverse section of the dorsal column close-up photomicrographs that are shown in Figures 1b and 1c.

Supplementary Figure 2

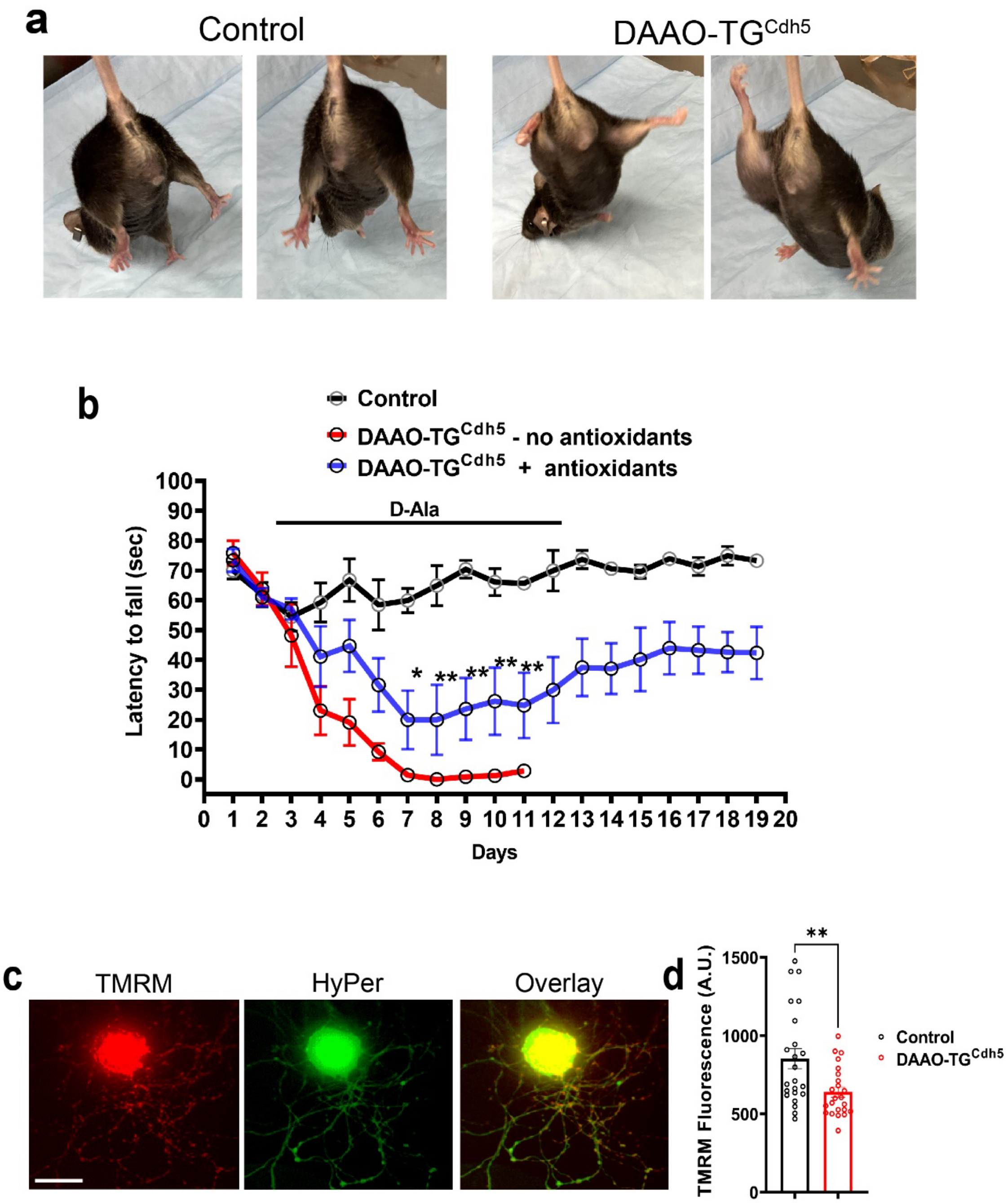

Supplementary Figure 2:

a shows a representative photographs of mice in a tail hanging test[2] in which DAAO-TG^{Cdh5} mice and littermate control Cre^{+}/TG^{-} mice were treated with D-alanine in the drinking water and tested after D-alanine feeding (0.75 M for 11 days). Individual mice were dangled by their tails for 30 seconds and observed in this standard behavioral assay, which tests for limb coordination and proprioception[3]. **b** shows the effects of antioxidants on ataxia development and recovery, as measured using the Rotarod test[4]. DAAO-TG^{Cdh5} mice were provided either with drinking water containing an antioxidant "cocktail" consisting of N-acetyl cysteine (10 mM) and sodium selenite (10 μM) or with regular water ("no antioxidants") for two days prior to the initiation of D-alanine feeding (D-alanine feeding is noted on the graph). Cre^{+}/TG^{-} control mice received D-alanine but no antioxidants. The Rotarod test was performed daily on all three groups, quantitating the latency to fall for each animal (n = 6 in each group). By 11 days, all of the DAAO-TG^{Cdh5} mice fed D-alanine without antioxidants need to be euthanized because of incapacitating ataxia, but the DAAO-TG^{Cdh5} mice fed antioxidants had a much less severe phenotype than the DAAO-TG^{Cdh5} mice without antioxidants. Data are presented as mean values +/- SEM; (* denotes $p < 0.05$ and ** denotes $p < 0.01$, two-way ANOVA). When D-alanine feeding was stopped, the mice fed antioxidants had a partial recovery assessed using the Rotarod test. D-alanine feeding had no effect whatsoever on the Cre^{+}/TG^{-} control mice. **c** shows representative photomicrographs of a DRG neuron cultured from a DAAO-TG^{Cdh5} mouse treated for 5 days with 0.75 M D-alanine and imaged for mitochondrial membrane potential (TMRM) or for GFP transgene expression (the GFP detects the YFP that is part of the DAAO-HyPer transgenic fusion protein), as noted. **d** shows pooled data quantitating the TMRM signal in cultured DRG neurons from D-alanine-fed DAAO-TG^{Cdh5} and control mice. Scale bars indicate 30 μm. n = 3 biologically independent mice each group and n =23 (experiments). Data are presented as mean values +/- SEM; ** denotes $p = 0.0053$, unpaired t-test.

Supplementary Figure 3

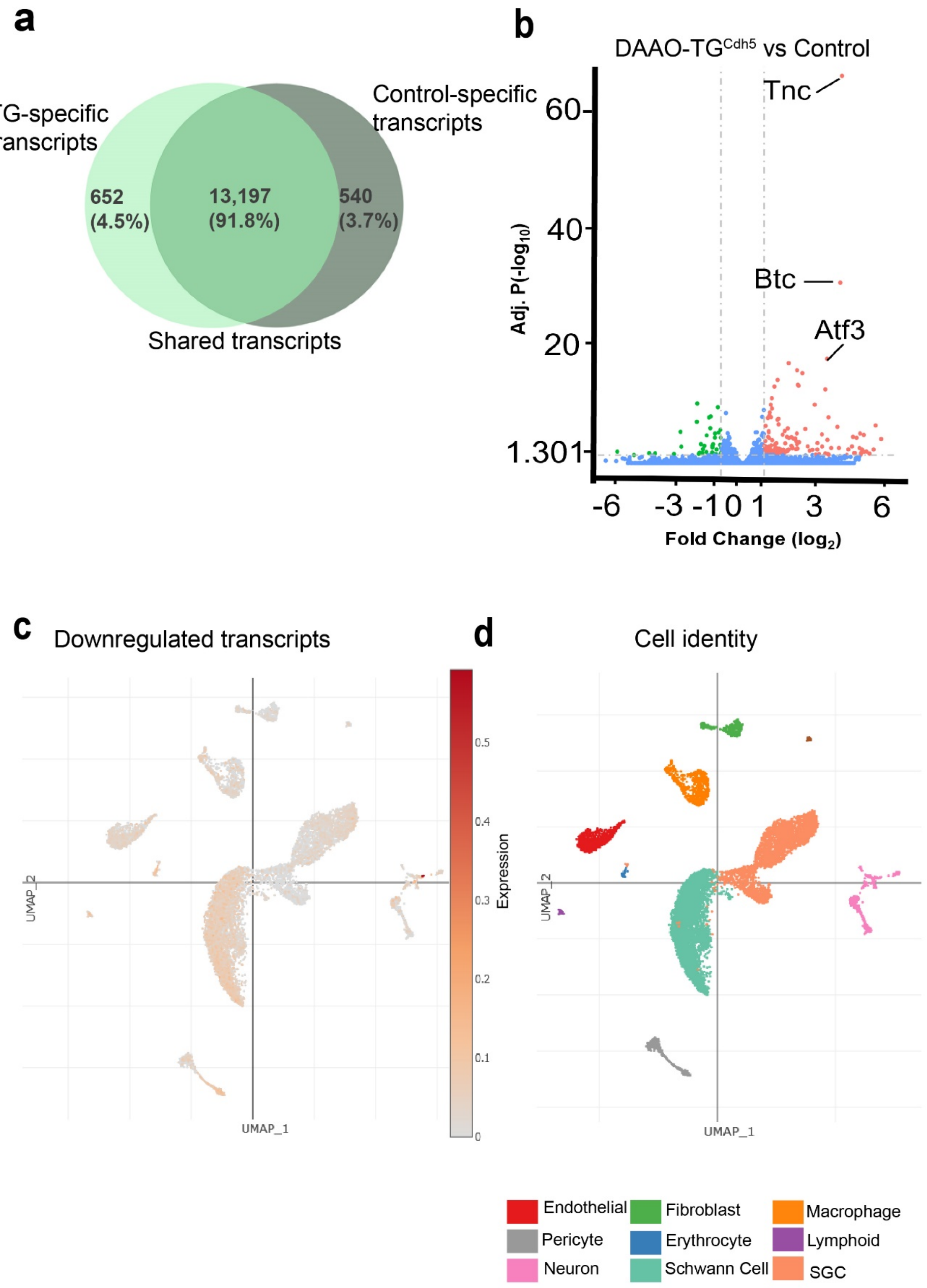

Supplementary Figure 3:

a shows a Venn diagram presenting the numbers of uniquely expressed genes present in DRG isolated from D-alanine treated DAAO-TG^{Cdh5} mice relative to the abundance of these transcripts in control littermates. **b** shows a volcano plot of the DRG transcripts of DAAO^{Cdh5} vs. Cre^{+}/TG^{-} littermate control groups. Gray line indicate fold change threshold was 2 (x-axis) and the p adj threshold was set at 0.05 (y-axis). Significantly downregulated and upregulated genes are noted in red; unchanged genes are shown in green. We note several significantly upregulated transcripts: tenascin C (*Tnc*), Betacellulin (Btc), and Activating Transcription Factor 3 (ATF3). **c** shows a uniform manifold approximation and projection (UMAP) plot representing the downregulated (≥ 2 fold) transcripts in DRG superimposed over the global uniform manifold approximation and projection distribution; significantly increased transcripts are noted in red. **d** shows the global uniform manifold approximation and projection plot of the different cell populations of the DRG. Each dot represents an individual cell, and the colors represent the cells' respective subcluster, as noted at the bottom of the figure. The cellular origin of the downregulated DRG transcripts can be imputed by the overlay of the bulk RNA sequencing data obtained in the present studies (**c**) with the recently-reported single-cell RNA sequencing data from Jager et al[5] published in the single cell portal (Single Cell Portal: https://singlecell.broadinstitute.org/single_cell) (**d**).

Supplementary Figure 4

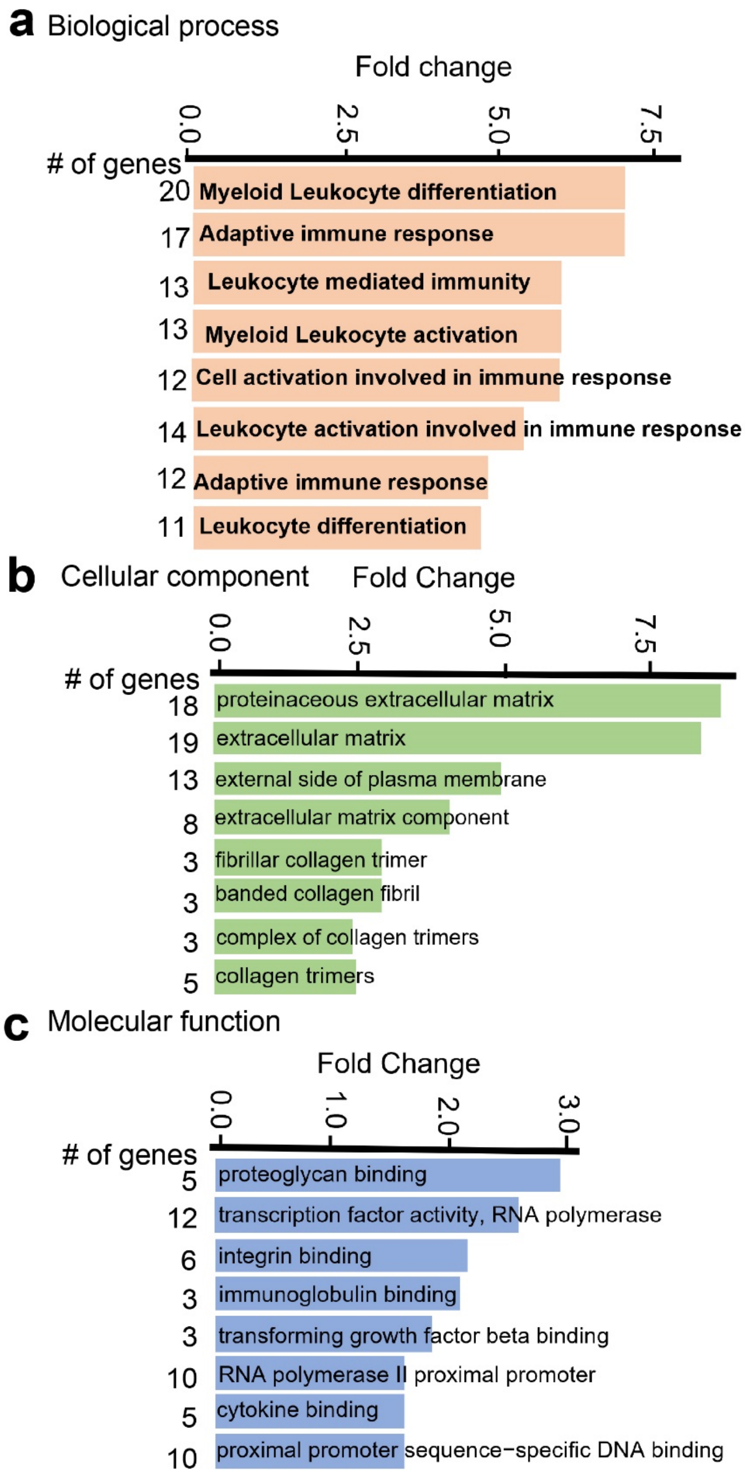

Supplementary Figure 4:

RNA sequencing was performed in DRG isolated from DAAO-TG^{Cdh5} mice or control Cre^{+}/TG^{-} littermates chronically treated with D-alanine (0.5 M for 7 weeks) and analyzed using Gene Ontology[6]**.** **a** shows GO analyses of biological functions/pathways. **b** shows GO analyses based on cellular components. **c** shows the GO analyses based on molecular function. The number of genes represented within each pathway are noted for each of the highly upregulated differentially expressed genes, which are grouped using clusterProfiler software (R package); the 30 most significant terms were selected for display.

Supplementary Figure 5

a

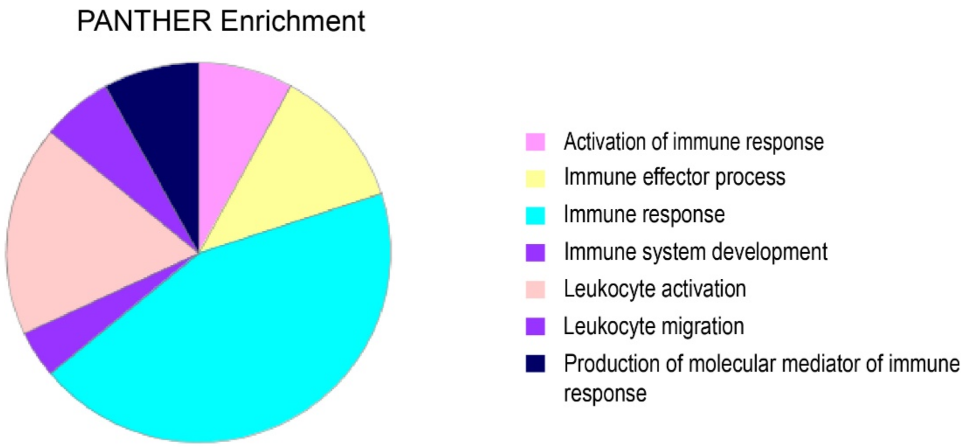

b

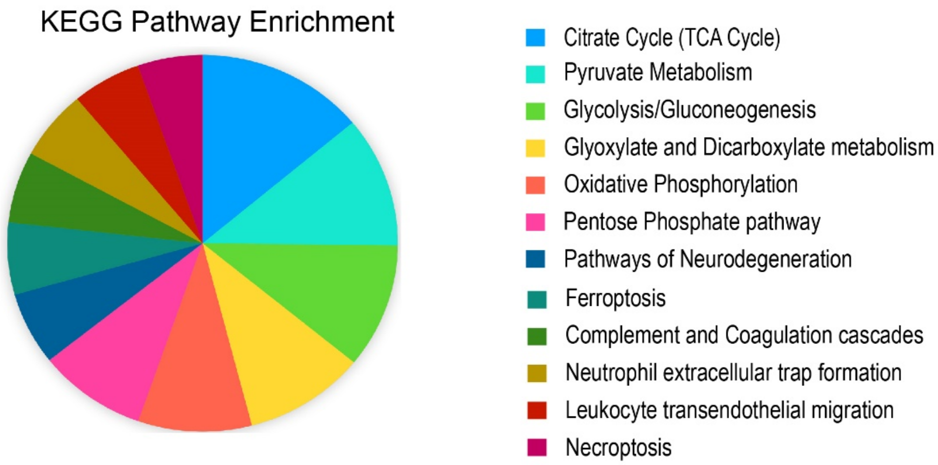

Supplementary Figure 5:
a presents a pie chart derived from Panther pathway enrichment analysis, which reveals a preponderance of immune response related pathways. Each sector is separately color coded, and the size of the sectors is proportional to the percent enrichment calculated with respect to the total enrichment score. **b** shows a pie chart presenting the major pathways enriched by KEGG pathway analysis reflecting the presence of important metabolic pathways as well as immune pathways. Each fraction has been separately color coded and percent enrichment was calculated with respect to total enrichment score.

Supplementary Figure 6:

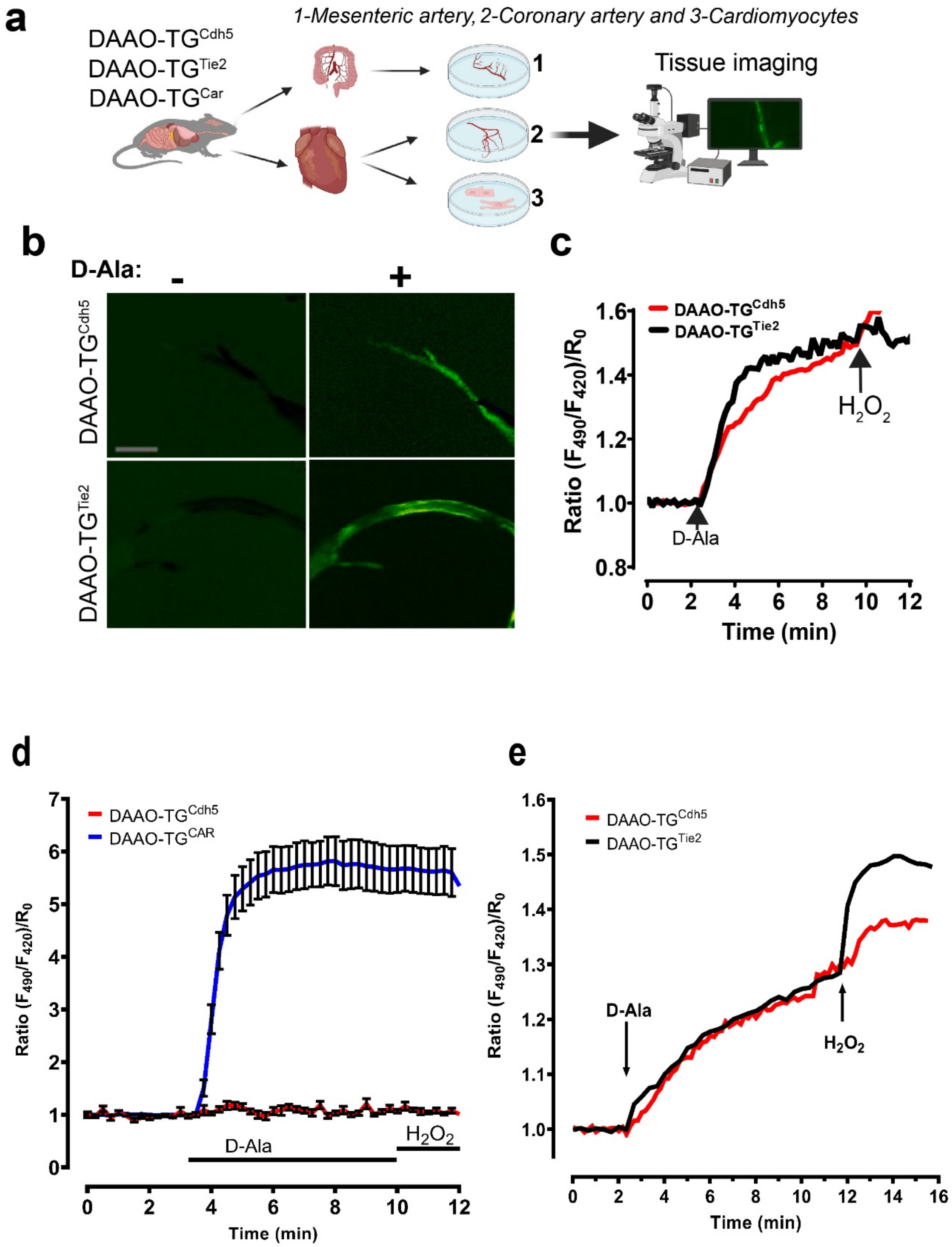

Supplementary Figure 6:
a shows a schematic of the experimental approach to generate (DAAO) and detect (HyPer biosensor) H_2O_2 in vascular and cardiac tissues isolated from three different DAAO-TG transgenic mouse lines. **b** shows representative live tissue HyPer ratiometric images of intact mesenteric arteries isolated from untreated DAAO-TG^{Cdh5} and DAAO-TG^{Tie2} mice before and 5 minutes after the addition of D-alanine to the arteries (10 mM), revealing a robust arterial HyPer response to D-alanine for both of these transgenic lines. **c** shows quantitation of the HyPer ratio from mesenteric arteries treated with 10 mM D-alanine and monitored in real time; when the D-alanine signal stabilized, exogenous H_2O_2 (25 μM) was added to the cells to assess for any additional HyPer response. Scale bars indicate 200 μm. The images are representative of n = 3 mice per group. **d** presents ratiometric data from cardiac myocytes isolated from untreated DAAO-TG^{Cdh5} and DAAO-TG^{Car} mice (the DAAO-TG^{Car} transgenic line expresses DAAO-HyPer in cardiac myocytes under control of the cardiac-specific Myh6 promoter). The isolated cardiac myocytes were imaged for H_2O_2 production following the addition of 10 mM D-alanine and then again in response to addition of H_2O_2 (25 μM); only the cardiac myocytes isolated from DAAO-TG^{Car} mice showed a response, whereas the cardiac myocytes isolated from DAAO-TG^{Cdh5} showed no signal whatsoever. Data are presented as mean values +/- SEM. Scale bars indicate 200 μm. **e** shows ratiometric data from coronary arteries isolated from the hearts of untreated DAAO-TG^{Cdh5} and DAAO-TG^{Tie2} mice. The coronary arteries were mounted on the microscope stage and the HyPer ratio was monitored in real time as D-alanine was added; when the signal reached a plateau, H_2O_2 (25 μM) was added to elicit a maximal HyPer signal. Both the DAAO-TG^{Cdh5} and DAAO-TG^{Tie2} lines show robust HyPer responses in mesenteric and coronary arteries, but only the DAAO-TG^{Car} line shows functional transgene expression in cardiac myocytes. These tracings are representative of n $\geq$ 3 mice per group.

Supplementary Figure 7:

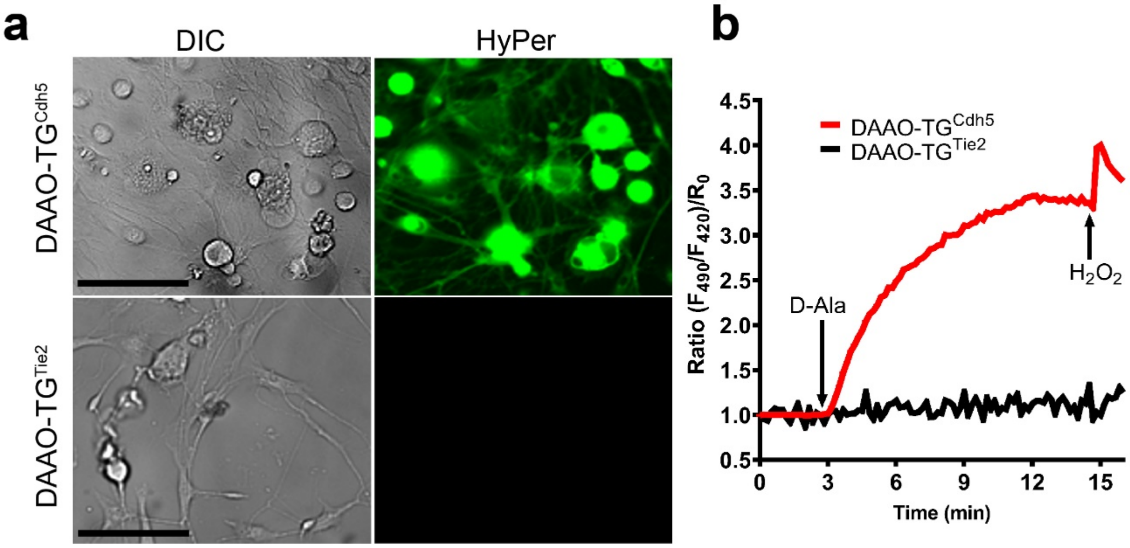

Supplementary Figure 7:
This figure shows representative images of cultured DRG neurons isolated from untreated DAAO-TG^{Cdh5} and DAAO-TG^{Tie2} mice. In **a**, DIC images are shown on the left, and HyPer ratiometric images are shown on the right after the addition of D-alanine (10 mM, 5 min) to the neurons. **b** shows quantitation of the live cell HyPer ratiometric signals from cultured DRG treated with 10 mM D-alanine and monitored in real time; a robust HyPer signal is seen in response to D-alanine for the DAAO-TG^{Cdh5} neurons, but there is no response in the DRG neurons isolated from DAAO-TG^{Tie2} mice. Scale bars indicate 200 μm. All images are representative of n = 3 mice per group.

Supplementary References

1. Chu, V. T. *et al.* Efficient generation of Rosa26 knock-in mice using CRISPR/Cas9 in C57BL/6 zygotes. *BMC Biotechnology* **16**, 1–15 (2016).
2. Miedel, C. J., Patton, J. M., Miedel, A. N., Miedel, E. S. & Levenson, J. M. Assessment of spontaneous alternation, novel object recognition and limb clasping in transgenic mouse models of amyloid-β and tau neuropathology. *Journal of Visualized Experiments* **2017**, 1–8 (2017).
3. Chen, X. J. *et al.* Proprioceptive sensory neuropathy in mice with a mutation in the cytoplasmic dynein heavy chain 1 gene. *Journal of Neuroscience* **27**, 14515–14524 (2007).
4. Nuber, S. *et al.* Abrogating Native α-Synuclein Tetramers in Mice Causes a L-DOPA-Responsive Motor Syndrome Closely Resembling Parkinson's Disease. *Neuron* **100**, 75–90 (2018).
5. Jager, S. E. *et al.* Comparative transcriptional analysis of satellite glial cell injury response. *Wellcome Open Research* **7**, 1–23 (2022).
6. Dobin, A. *et al.* STAR: Ultrafast universal RNA-seq aligner. *Bioinformatics* **29**, 15–21 (2013).
