# Supplementary material for: Sensory ataxia and cardiac hypertrophy caused by neurovascular oxidative stress in chemogenetic transgenic mouse lines
Source: Nat Commun. 2023 May 29;14:3094. doi: 10.1038/s41467-023-38961-0 (PMC10227029; doi:10.1038/s41467-023-38961-0)
Supplement: Supplementary file 3 — Description to Additional Supplementary Information [file 41467_2023_38961_MOESM3_ESM.pdf]

Description of Additional Supplementary Files

Supplementary Video 1: Ataxia in a DAAO-TG^{Cdh5} transgenic mouse fed D-alanine The transgenic mouse filmed in this video had been fed 0.75M D-alanine in its drinking water for 3 days. The locomotory disturbance seen here was observed in all DAAO-TG^{Cdh5} transgenic animals that were fed D-alanine, but not in any of the D-alanine-fed Cre^+/TG^- littermate control animals nor in the DAAO-TG^{Cdh5} mice
