# Supplementary material for: Sensory ataxia and cardiac hypertrophy caused by neurovascular oxidative stress in chemogenetic transgenic mouse lines
Source: Nat Commun. 2023 May 29;14:3094. doi: 10.1038/s41467-023-38961-0 (PMC10227029; doi:10.1038/s41467-023-38961-0)
Supplement: Supplementary file 5 — Reporting Summary [file 41467_2023_38961_MOESM5_ESM.pdf]

nature portfolio

Corresponding author(s): *Double-blind peer review submissions: write DBPR and your manuscript number here instead of author names.*

Last updated by author(s): May 23, 2023

Reporting Summary

Nature Portfolio wishes to improve the reproducibility of the work that we publish. This form provides structure for consistency and transparency in reporting. For further information on Nature Portfolio policies, see our Editorial Policies and the Editorial Policy Checklist.

Statistics

For all statistical analyses, confirm that the following items are present in the figure legend, table legend, main text, or Methods section.

n/a	Confirmed	
☐	☒	The exact sample size (*n*) for each experimental group/condition, given as a discrete number and unit of measurement
☐	☒	A statement on whether measurements were taken from distinct samples or whether the same sample was measured repeatedly
☐	☒	The statistical test(s) used AND whether they are one- or two-sided<br>*Only common tests should be described solely by name; describe more complex techniques in the Methods section.*
☐	☒	A description of all covariates tested
☐	☒	A description of any assumptions or corrections, such as tests of normality and adjustment for multiple comparisons
☐	☒	A full description of the statistical parameters including central tendency (e.g. means) or other basic estimates (e.g. regression coefficient) AND variation (e.g. standard deviation) or associated estimates of uncertainty (e.g. confidence intervals)
☐	☒	For null hypothesis testing, the test statistic (e.g. *F*, *t*, *r*) with confidence intervals, effect sizes, degrees of freedom and *P* value noted<br>*Give P values as exact values whenever suitable.*
☒	☐	For Bayesian analysis, information on the choice of priors and Markov chain Monte Carlo settings
☒	☐	For hierarchical and complex designs, identification of the appropriate level for tests and full reporting of outcomes
☒	☐	Estimates of effect sizes (e.g. Cohen's *d*, Pearson's *r*), indicating how they were calculated

Our web collection on statistics for biologists contains articles on many of the points above.

Software and code

Policy information about availability of computer code

Data collection	Olympus IX81Microscope with an ImagEM CCD camera (Hamamatsu) with Metafluor Software (Molecular Devices), JEOL 1200EX electron microscope and an AMT 2k CCD camera.
Data analysis	GeneCodis and Panther software packages to characterize pathway enrichment,Vevo LAB software (V.3.1.1 FUJIFILM Visualsonics, Toronto, Canada), GraphPad Prism 11.0 (GraphPad Software, La Jolla, CA), and ClusterProfiler software (R package). R version 4.2.1

For manuscripts utilizing custom algorithms or software that are central to the research but not yet described in published literature, software must be made available to editors and reviewers. We strongly encourage code deposition in a community repository (e.g. GitHub). See the Nature Portfolio guidelines for submitting code & software for further information.

Data

Policy information about availability of data

All manuscripts must include a data availability statement. This statement should provide the following information, where applicable:

- Accession codes, unique identifiers, or web links for publicly available datasets
- A description of any restrictions on data availability
- For clinical datasets or third party data, please ensure that the statement adheres to our policy

Data supporting the findings of this study are available in the article and its Supplementary information. Source data are provided with this paper. Source Data file has also been deposited in figshare under accession code https://doi.org/10.6084/m9.figshare.22803020. The raw data and processed data for bulk RNA-seq of DRG

neuronal cells were deposited in Genome Sequence Archive with accession ID GSE229143 (https://www.ncbi.nlm.nih.gov/geo/query/acc.cgi?acc=GSE229143). The list of genes that were significantly upregulated or downregulated in DAAO-TGCdh5 mice compared with littermate control was analyzed using the Single Cell Portal and overlayed on the single cell RNA sequencing data (https://singlecell.broadinstitute.org/single_cell/study/SCP1539/dorsal-root-ganglia-cells-after-nerve-injury) reported by Jager et al18. Raw data related to mouse genetic resources can be obtained from the NCBI Repository by requesting and following the guidelines for Genome Sequence Archive for noncommercial use GSE229143 (https://www.ncbi.nlm.nih.gov/geo/query/acc.cgi?acc=GSE229143) . The mass spectrometry proteomics data have been deposited to the ProteomeXchange Consortium via the PRIDE66 partner repository with an accession ID PXD041382 (https://www.ebi.ac.uk/pride/archive/projects/PXD041382).

Human research participants

Policy information about studies involving human research participants and Sex and Gender in Research.

Reporting on sex and gender	Not Applicable
Population characteristics	Not Applicable
Recruitment	Not Applicable
Ethics oversight	Not Applicable

Note that full information on the approval of the study protocol must also be provided in the manuscript.

Field-specific reporting

Please select the one below that is the best fit for your research. If you are not sure, read the appropriate sections before making your selection.

☒ Life sciences ☐ Behavioural & social sciences ☐ Ecological, evolutionary & environmental sciences

For a reference copy of the document with all sections, see nature.com/documents/nr-reporting-summary-flat.pdf

Life sciences study design

All studies must disclose on these points even when the disclosure is negative.

Sample size	Sample size was chosen based on our previous publication (Steinhorn, B. et al. 2018)
Data exclusions	No data was excluded.
Replication	To gain meaningful statistical differences, n≥6 animal per group were used in behavior test (Open field test, rotarod test and grip strength test), n≥9 mice per group were used in in vivo cardiac function analysis (Echocardiography), and n≥ 3 animal per group were used in all individual in vitro experiments, as indicated in the figure legends.
Randomization	All mice were randomly grouped to avoid selection bias.
Blinding	All experiments have been performed by blinded observers.

Reporting for specific materials, systems and methods

We require information from authors about some types of materials, experimental systems and methods used in many studies. Here, indicate whether each material, system or method listed is relevant to your study. If you are not sure if a list item applies to your research, read the appropriate section before selecting a response.

Materials & experimental systems

n/a	Involved in the study	
☐	☒	Antibodies
☒	☐	Eukaryotic cell lines
☐	☐	Palaeontology and archaeology
☐	☒	Animals and other organisms
☒	☐	Clinical data
☒	☐	Dual use research of concern

Methods

n/a	Involved in the study	
☒	☐	ChIP-seq
☒	☐	Flow cytometry
☒	☐	MRI-based neuroimaging

Antibodies

Antibodies used	The following primary antibodies were used: GFP (cat. no. 2956, clone no. D5.1, Dilution factor 1:1000, Cell Signaling Technology), Cdh5 (cat. no. sc9989, lot no. c1622, clone no. F-8, dilution factor 1:200 Santa Cruz Biotechnology), GAPDH (cat. no 2118, lot. No. 16, clone no. 14C10, dilution factor 1:2000, Cell Signaling Technology), NeuN (cat. no. mab377, lot no. 382727, dilution factor 1:100 Millipore), and Tyrosine Hydroxylase (cat. no. AB152, lot. No. 3845256, clone no. is not available, dilution factor 1: 1000, EMD, Millipore)
Validation	GFP: https://www.cellsignal.com/products/primary-antibodies/gfp-d5-1-rabbit-mab/2956?site-search-type=Products&N=4294956287&Ntt=2956s&fromPage=plp&_requestid=1043717<br>GAPDH: https://www.cellsignal.com/products/primary-antibodies/gapdh-14c10-rabbit-mab/2118<br><br>Cdh5: https://www.scbt.com/p/ve-cadherin-antibody-f-8?requestFrom=search(used in this article:https://pubmed.ncbi.nlm.nih.gov/36735487/).<br><br>NeuN: https://www.emdmillipore.com/US/en/product/Anti-NeuN-Antibody-clone-A60,MM_NF-MAB377<br><br>TH Anti-Tyrosine Hydroxylase:https://www.emdmillipore.com/US/en/product/Anti-Tyrosine-Hydroxylase-Antibody,MM_NF-AB152

Palaeontology and Archaeology

Specimen provenance	N/A
Specimen deposition	N/A
Dating methods	N/A

☐ Tick this box to confirm that the raw and calibrated dates are available in the paper or in Supplementary Information.

Ethics oversight	N/A

Note that full information on the approval of the study protocol must also be provided in the manuscript.

Animals and other research organisms

Policy information about studies involving animals; ARRIVE guidelines recommended for reporting animal research, and Sex and Gender in Research

Laboratory animals	Equal numbers of male and female mice were studied, and 6-8 animals of each sex were analyzed for each experimental treatment and genotype, except as noted in the figure legends. Sex was not studied as a biological variable because our initial observations indicated that both male and female DAAO-TGCdh5 transgenic mice develop ataxia after D-alanine feeding, and the effect(s) of sex on the phenotype were not further characterized in these studies. Data disaggregated for sex are provided in the source data file. Studies were commenced when the animals were 8-12 weeks of age. A transgenic conditionally activatable HyPer-DAAO construct was made in collaboration with Novartis by cloning a new stop codon flanked by loxP sites into the 5'-coding region of the cDNA encoding the HyPer-DAAO fusion protein (the nucleotide sequence is in reference7) downstream of the CAG promoter (see Supplementary Figure 1). Using this approach, synthesis of HyPer-DAAO protein is blocked at the "floxed" stop codon until expression of Cre recombinase excises the stop codon and permits transcription of the full-length transgene. This construct was directed to the Rosa26 locus by CRISPR/Cas9 methods and transgenic founder lines were generated in C57/Bl6 mice using standard methods; founder lines were identified by PCR, confirming insertion of a single copy of the intact transgenic construct into the Rosa26 locus. This DAAO-TGLoxP mouse was then crossed with mice expressing Cre recombinase under control of the endothelial cell-specific Cdh5 promoter (strain 033055, Jackson Labs). DAAO-TGCdh5 positive offspring were identified by PCR, and are maintained in a C57/Bl6 background. Littermates containing Cre but lacking the transgene (Cre+/TG-) served as controls in order to control for possible off-target effects from Cre recombinase expression49. We generated a second transgenic mouse line DAAO-TGTie2 by crossing the DAAO-TGLoxP mouse with a commercially available mouse line (Jackson Labs strain 008863) that expresses Cre recombinase under control of the endothelial cell-specific Tie2 promoter. A third mouse line was also generated in collaboration with Novartis in which the DAAO-HyPer transgene is under control of the cardiac-specific Myh6 promoter to yield the DAAO-TGCar line . All strains are maintained on the C57BL/6 background.
Wild animals	There was no wild animal used in the study.
Reporting on sex	Equal numbers of male and female mice were studied. Data disaggregated for sex have been reported in the source data file.
Field-collected samples	There were no field collected samples used in the study.

Ethics oversight	All animal experiments were carried out under NIH guidelines for the care of laboratory mice, and all animal protocols were approved by the Brigham and Women's Hospital Institutional Animal Care and Use Committee (protocol 2016N000278).

Note that full information on the approval of the study protocol must also be provided in the manuscript.
