# Supplementary material for: Sensory ataxia and cardiac hypertrophy caused by neurovascular oxidative stress in chemogenetic transgenic mouse lines
Source: Nat Commun. 2023 May 29;14:3094. doi: 10.1038/s41467-023-38961-0 (PMC10227029; doi:10.1038/s41467-023-38961-0)
Supplement: Supplementary file 6 — Source Data [file 41467_2023_38961_MOESM6_ESM.zip › Source Data/Source Supplementary information.pptx]

## Slide 1
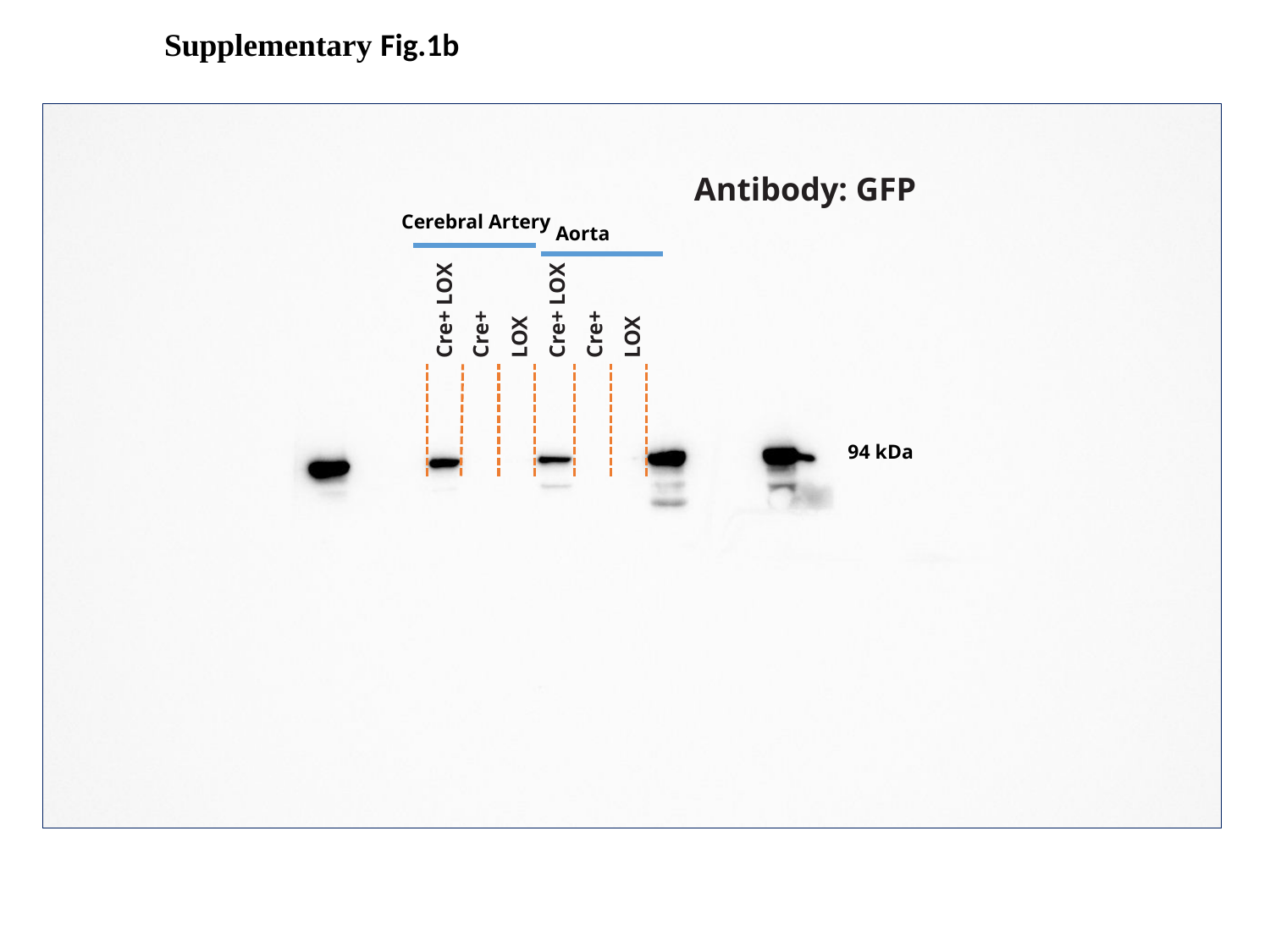

Supplementary Fig.1b
Antibody: GFP
Cerebral Artery
Aorta
Cre+ LOX
Cre+ LOX
 LOX
 LOX
Cre+
Cre+
94 kDa

## Slide 2
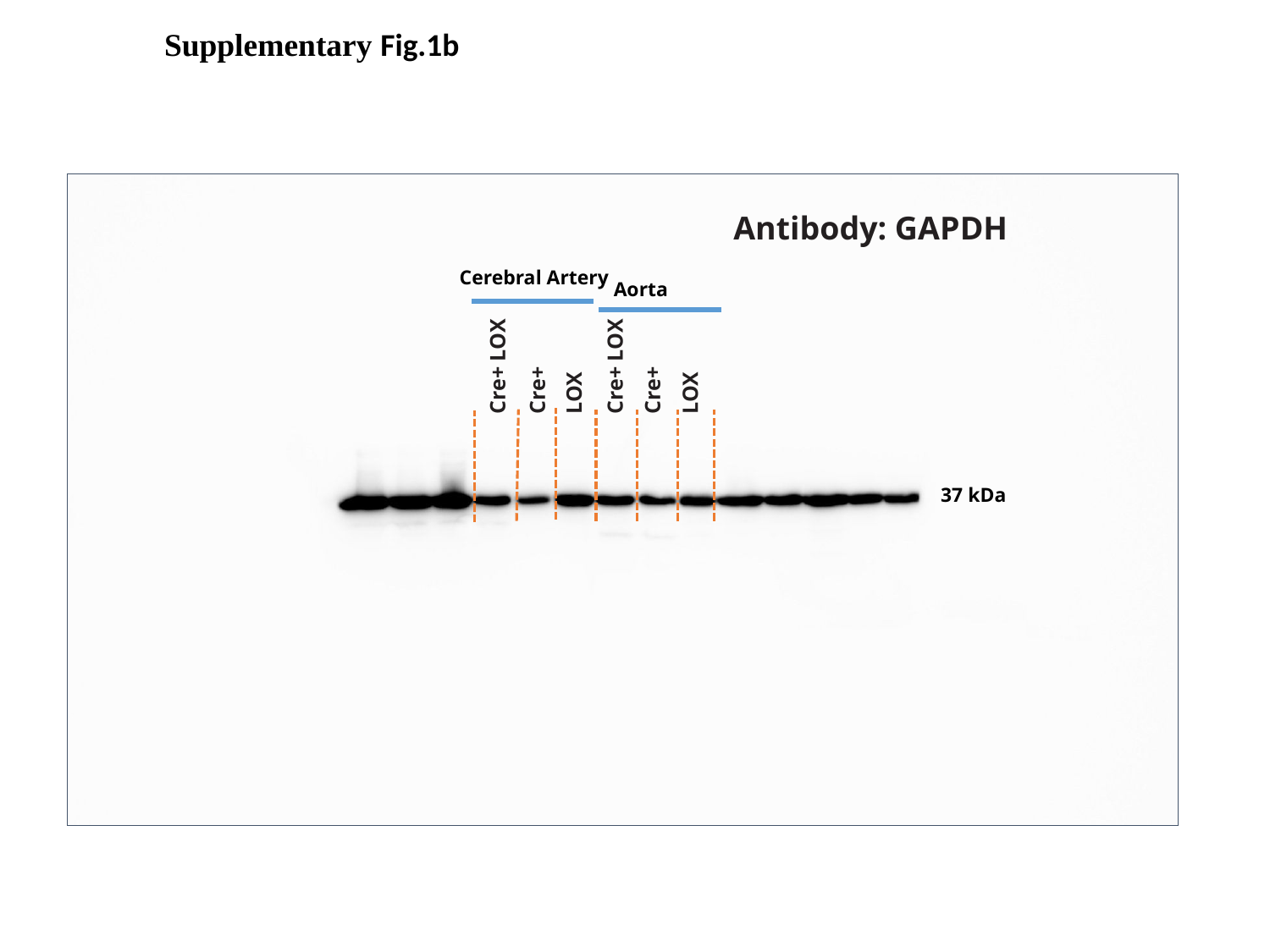

Supplementary Fig.1b
Antibody: GAPDH
Cerebral Artery
Aorta
Cre+ LOX
Cre+ LOX
 LOX
 LOX
Cre+
Cre+
37 kDa

## Slide 3
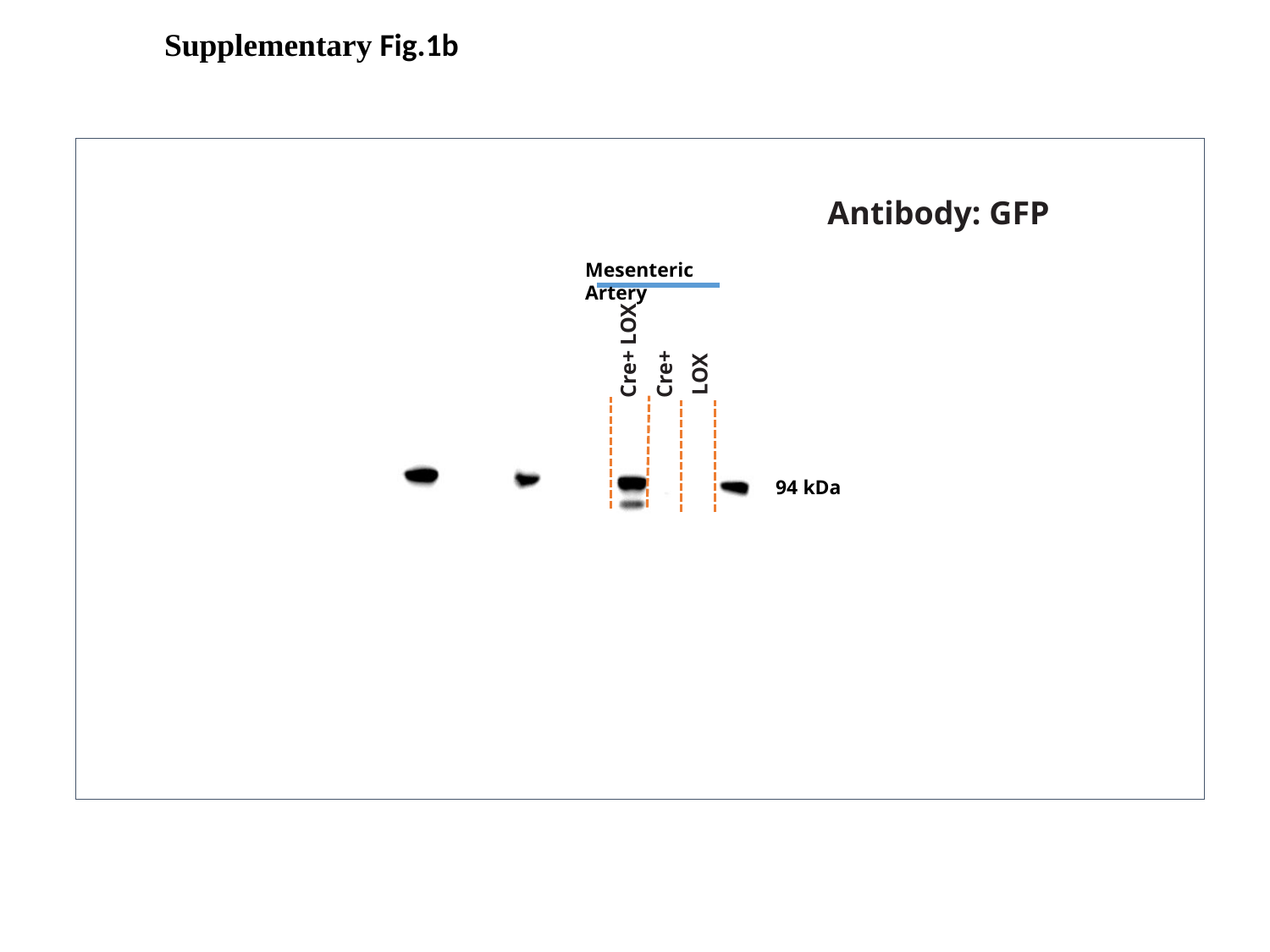

Supplementary Fig.1b
Antibody: GFP
Mesenteric Artery
Cre+ LOX
 LOX
Cre+
94 kDa

## Slide 4
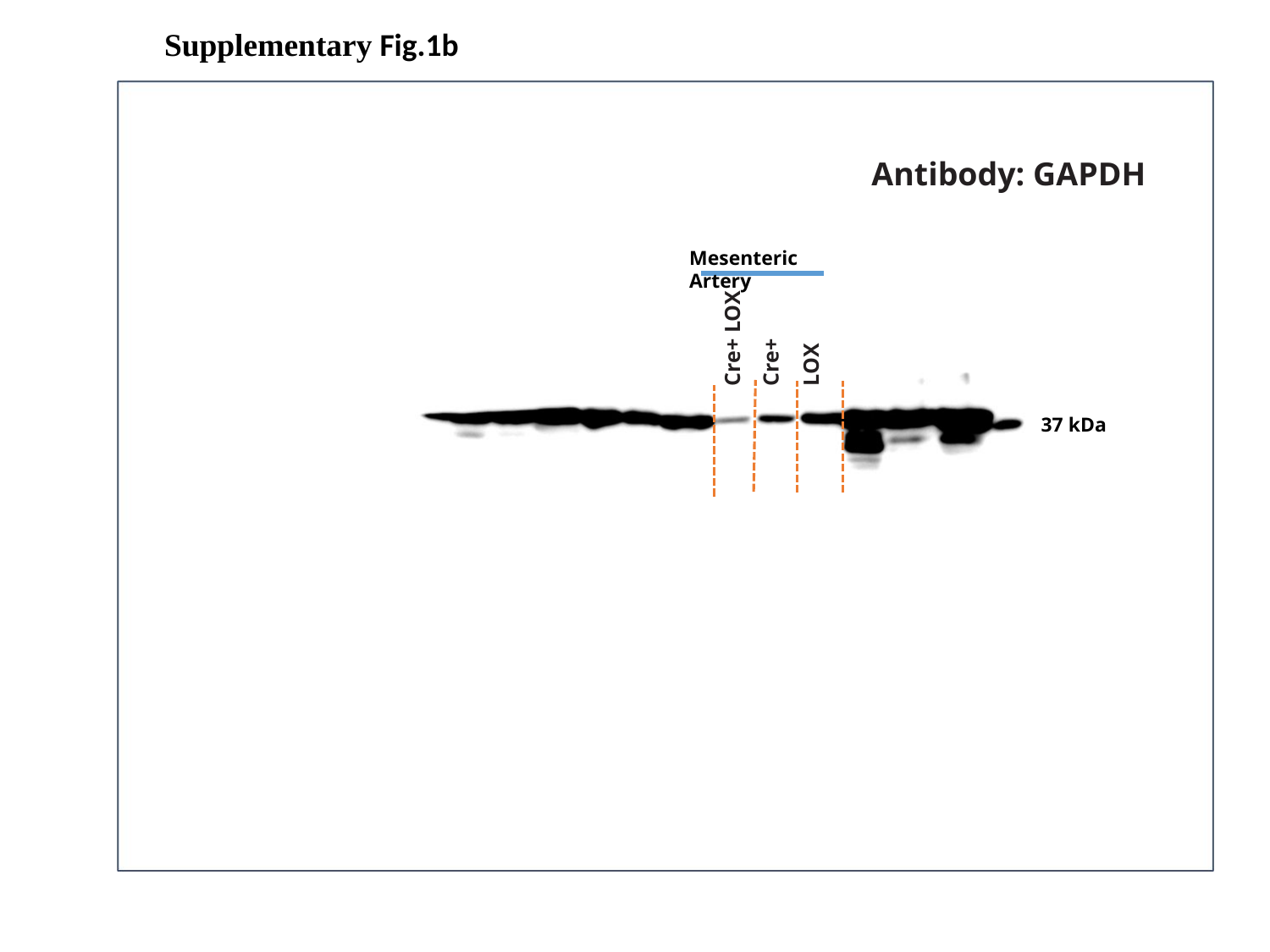

Supplementary Fig.1b
Antibody: GAPDH
Mesenteric Artery
Cre+ LOX
 LOX
Cre+
37 kDa
